# Does Meta-induction Justify Induction: Or Maybe Something Else?

**DOI:** 10.1007/s10838-022-09620-7

**Published:** 2023-02-22

**Authors:** J. Brian Pitts

**Affiliations:** 1 University of Cambridge, Cambridge, UK; 2 University of Lincoln, Lincoln, UK; 3 University of South Carolina, Columbia, SC USA

## Abstract

According to the Feigl–Reichenbach–Salmon–Schurz pragmatic justification of induction, no predictive method is guaranteed or even likely to work for predicting the future; but if anything will work, induction will work—at least when induction is employed at the meta-level of predictive methods in light of their track records. One entertains a priori all manner of esoteric prediction methods, and is said to arrive a posteriori at the conclusion, based on the actual past, that object-level induction is optimal. Schurz’s refinements largely solve the notorious short-run problem. A difficulty is noted, however, related to short-run worries but based on localized disagreement about the past, a feature characteristic of real debates (especially early modern) involving induction in intellectual history. Given the evidence about past events, *unfiltered by induction*, meta-induction might support a partly non-inductive method—especially as judged by proponents of esoteric prediction methods, who presumably believe that their methods have worked. Thus induction is justified meta-inductively in contexts where it was uncontroversial, while not obviously justified in key contexts where it has been disputed. This objection, momentarily sensed by Reichenbach regarding clairvoyance, is borne out by the Stoics’ use of meta-induction to justify both science and divination and by ancient Hebrew examples of meta-induction. Schurz’s recently introduced criteria for acceptance of testimony play a crucial role in arriving at object-level induction using meta-induction, but one might question them. Given the need for judgment in accepting testimony, it is unclear that the subjectivity of Howson’s Bayesian answer to Hume’s problem is overcome.

## Introduction

Partly because the logical empiricists renounced rationalistic a priori knowledge claims such as (neo-)Kantianism, it became important to wrestle seriously with inductive skepticism à la Hume. Hans Reichenbach’s book *Experience and Prediction* (Reichenbach 1938) was an important contribution, advancing a “pragmatic” justification of induction. He emphasized the importance of the problem:

Inductive inference cannot be dispensed with because we need it for the purpose of action […]. I should say a philosopher who is to put aside his principles any time he steers a motorcar is a bad philosopher. (Reichenbach 1938, 346–347)

Presumably Reichenbach’s point holds *mutatis mutandis* for bicycling and taking public transport.

Efforts to justify induction attracted considerable attention (Kyburg 1964) into the 1970s, when they dwindled presumably due to exhaustion rather than completion. Salmon (1955; 1957; 1966; 1963) and John Clendinnen (1966; 1977; 1982; 1986) made efforts when little other work was occurring. Salmon wondered, in the absence of a solution, “[w]hy not turn to voodoo, which would be simpler, cheaper, less time consuming, and more fun?” (Salmon 1966, 55). Quine agreed that “telepathy and clairvoyance are scientific options, however moribund. It would take some extraordinary evidence to enliven them,” but it could happen (Quine 1992). In largely the same period that efforts to justify induction went into eclipse, Bayesianism gained popularity among philosophers of science. Generally this Bayesianism has tended toward a subjective form (Howson et al. 1993). Indeed objective Bayesianism had become rare among philosophers prior to Jon Williamson’s work (Williamson 2010).

Colin Howson devoted an entire book to the problem of induction, written from a subjective Bayesian perspective (Howson 2000). He noted that philosophers generally agree that Hume’s problem cannot be solved; but rather than drawing interesting consequences, they promptly forget this fact when thinking of other matters and proceed as if induction were in fact clearly justified (Howson 2000, 1–2). Howson urged that philosophers take their work on Hume’s problem of induction seriously enough to draw the appropriate radical conclusions. Induction is either unjustifiable or already justified by Bayesian confirmation theory, depending on one’s opinions, because one has to supply one’s own inductive assumptions.

But what I do believe, and I believe that this extended footnote to Hume shows, is that no theory of rationality that is not entirely question-begging can tell us what it is rational to believe about the future, whether based on what the past has displayed or not. This is not to say that evidence tells us nothing. The trouble is that what it does tell us cannot be unmixed from what we are inclined to let it tell us. Increasing observational data certainly, *provably, *reinforces some hypotheses at the expense of others, but only if we let it by a suitable assignment of priors. (Howson 2000, 239–240, emphasis in the original)

Bayesianism, though a fully adequate inductive logic in a certain sense on Howson’s view, of itself does not license any specific way of generalizing from observed to unobserved instances. However, people who have beliefs about which sorts of generalizations are likely appropriate (as we all do, though perhaps not exactly the same ones) can use Bayesianism to draw specific justified conclusions. It is an inductive framework (Strevens 2004); it does not by itself contain the sort of detailed answers to which Carnap aspired for an inductive logic to give, even as he moved away from expecting a unique answer (Carnap 1952).

Clearly Howson’s proposal is one way of taking Hume’s problem seriously. It also has the virtue of being Bayesian. Bayesianism has ceased to be a mere philosophical darling and is now widely employed in various sciences (Hobson et al. 2010; Gregory 2005; Buck et al. 1996; Buck and Meson 2015), including even parts of biology and archaeology as well as physics and engineering. Hence one might expect to see increasing success in applying Bayesianism to real scientific problems. Howson’s answer is, however, relativist. Rorty and Boghossian debated relativism in the context of worldview-related epistemological differences of case of Galileo *vs*. Bellarmine (Rorty 1979; Paul 2006). If Bayesianism is as much the logic of science as Howson thought, then Rorty’s side of such debates might become formidable. Howson, writing after most people had quit working on the pragmatic justification proposal by Reichenbach and before Schurz’s work, had little to say about that tradition.

In recent years Gerhard Schurz (with occasional collaborators Paul Thorn and Eckhart Arnold) has mounted an improved “meta-inductive” defense of induction in the spirit of some of Reichenbach’s remarks (Schurz 2008; 2009; Schurz and Thorn 2016; Schurz et al. 2017; Schurz 2018; 2019; Schurz and Thorn 2020). The traditional pragmatic justification of induction admitted that induction cannot be shown to be either certain or likely to work, but claimed to show that induction is at least as likely to work as any alternative predictive scheme and hence is worthy of acceptance as the best bet, roughly speaking. While this flavor remains, Schurz’s meta-inductive justification is provably optimal in a well defined sense, with good control over the short run, a notorious weakness in Reichenbach’s work. This justification initially operates at a higher meta-inductive level, by a comparison of the track records of rival predictive methods (as opposed to object-level induction, some notion of the uniformity of nature), such that whatever predictive method has worked best so far, is vindicated. Thus input from known history is required. The question of what history is known, however, yields difficulties to which this paper is devoted. Vindication, crucially, is not an inductive argument (Feigl 1961; 1950), but only accepting the best bet (with no claim of certain or likely success), so the meta-inductive justification remains pragmatic and noncircular (Schurz 2019, 80), distinct from inductive justifications of induction pursued by Max Black among others (Black 1954), which Schurz rejects (Schurz 2019, 18–22). Vindication is rather a justification of an action, the adoption of a means of prediction.

A key idea of this paper, that in principle some esoteric method of prediction might be vindicated, depending on history, was clear in principle to Reichenbach:

Imagine a clairvoyant who is able to foretell the value *p* of the limit [of the relative frequency] in such an early stage of the series; of course we should be very glad to have such a man at our disposal. We may, however, without knowing anything about the predictions of the clairvoyant, make two general statements concerning them: (1) The indications of the clairvoyant can differ, if they are true, only in the beginning of the series, from those given by the inductive principle. In the end there must be an asymptotical convergence between the indications of the clairvoyant and those of the inductive principle. This follows from the definition of the limit. (2) The clairvoyant might be an imposter; his prophecies might be false and never lead to the true value *p* of the limit.

The second statement contains the reason why we cannot admit clairvoyance without control. How gain [*sic*] such control? It is obvious that the control is to consist in an application of the inductive principle: we demand the forecast of the clairvoyant and compare it with later observations; if then there is a good correspondence between the forecasts and the observations, we shall infer, by induction, that the man’s prophecies will also be true in the future. Thus it is the principle of induction which is to decide whether the man is a good clairvoyant. This distinctive position of the principle of induction is due to the fact that we know about its function of finally leading to the true value of the limit, whereas we know nothing about the clairvoyant. (Reichenbach 1938, 353–354)

The advantages of handy access to a “benevolent and semi-omniscient demigod” in choosing an optimal inductive method are evident (Festa 1993). While Reichenbach was not particularly clear about distinguishing levels of induction involved, Skyrms clarified such matters (Skyrms 1986, 44–46). Reichenbach seems not to have worried that this possibility could require any serious consideration of evidence, however.

It is instructive to compare the views of empiricist Reichenbach with the rationalist (and idealist) F. H. Bradley in order to see how the problem of induction is bound up what one takes to be historical events and hence what a Reichenbach-style view should regard as the historical track record. If induction is not known in advance to be reliable (as Reichenbach holds), then miracle-like events are not known in advance to be impossible or even highly improbable: dead people might cease being dead, for example. It is therefore unclear on what grounds one can exclude miracle reports from history, at least as data potentially to be taken seriously, as rationalists in philosophy and theology have been doing since Spinoza, Hume (in his critique of miracles) and Schleiermacher. Bradley is another good example. According to him, properly “critical” history presupposes the uniformity of nature and uses that presupposition to sift testimonial evidence, thus excluding reported miracles (Bradley 2011). Clearly Reichenbach would not be justified in using a track record sifted in accord with Bradley’s principles. Bradley had no more use for miracle claims than Reichenbach (or Schurz), but his rationalism contrasts sharply with their empiricism. For Bradley, external confirmation of a revelation by miracles is primitive superstition or anachronism (Bradley 1885). But for Reichenbach, a predicted miracle-type event (say, a dead person’s ceasing to be dead) is not known in advance to be impossible, and would provide some reason to take more seriously the predictor’s further predictions if it occurred—witness the remark on clairvoyance above—which is somewhat akin to a revelation confirmed by miracles.^1^ Hence where Bradley finds primitive superstition, Reichenbach could find good meta-inductive reasoning, though Reichenbach presumably thought that examples were scarce or non-existent *a posteriori*. How Reichenbach could know that such examples are scarce *a posteriori* is not very obvious, because reading history sifted by Bradley’s principles (as much respectable history by then had been, a trend that continued (Vincent 1995, 13–14)) omits putative evidence using *a priori* principles that Reichenbach rejected. Bradley presumably would think that Reichenbach’s logical empiricist reasoning overlaps significantly with primitive superstition or anachronism, differing only in fact-like historical claims. Bradley might wonder how Reichenbach could manage to sift the historical record and exclude miracle reports without already knowing or at least presupposing the uniformity of nature, thus invoking a priori knowledge that Reichenbach denied that we have. Hence easy assurance that meta-induction justifies (or vindicates) object-level induction *a posteriori* should not be so easy, while easy dismissal of miracle reports should not be so easy for those who aspire to be empiricists. This point will be further developed in response to Schurz and bears some relation to C. D. Broad’s identification of the tension between Hume’s critique of miracles and his induction skepticism (Broad 1916).

Schurz further develops Reichenbach’s empiricism, refreshingly attending to the beliefs of non-philosophers to apply to the real world what has traditionally been primarily an academic exercise conducted in a cultural vacuum.

It seems that so far, none of these attempts has been successful in giving a *positive *justification of induction, which establishes in a *noncircular *manner that the inductive method is a superior prediction method in terms of its success frequencies. Let me emphasize that this is neither obvious nor guaranteed. Millions of people *do *in fact believe in superior noninductive methods, be it God-guided inner intuition, clairvoyance, or other supernatural abilities. (Schurz 2008, 280, emphasis in the original; see also Schurz 2019, 16)

If Schurz is correct that none of these attempts had succeeded before the 2000s, then it would seem that the Enlightenment occurred around 250-odd years before its justification arrived. Perhaps those who held Enlightened opinions prior to the 2000s were holding just that, opinions? Be that as it may, in speaking of “millions,” Schurz appears actually to understate the case by a couple of orders of magnitude. By some counts more than half the world’s population is Christian, Muslim, or Hindu. The first two groups traditionally expect a final Judgment Day with a general resurrection, at which all dead people will cease to be dead and henceforth remain alive forever, for good or ill. Such an expectation is clearly contrary to object-level induction, according to which dead people remain dead (with remarkably few exceptions ever even claimed). Hinduism (apart perhaps from ‘purer’ philosophical versions which presumably are not so popular) seems seasoned with small-scale supernatural reports (e.g., Yogananda 1946). Traditional animists also believe generously in causally active spiritual beings, in some cases convincing western anthropologists who participate with witchdoctors (Stoller and Olkes 1987; Turner 2012). Correctly or not, billions of people believe in selectively superior (by their reckoning) non-inductive methods. Of course no one believes in a non-inductive method full-time, or ever has done so, or ever could. The fact that philosophers’ discussions have traditionally involved induction, counter-induction, randomness, and evil demons (Burks 1953), while neglecting the historically-culturally relevant rival of induction qualified by occasional exceptions for some alternative “esoteric” method (to use Schurz’s term), is curious if relevance to real disagreements, current or historical, was intended. Schurz is commendable for emphasizing real-world relevance, a point that this paper further develops.

On the other hand, physicists proverbially study a spherical cow not because they think that cows are spherical or nearly so, but because the model is tractable and it is better to do what one can than to do nothing at all, as long as one doesn’t confuse the model with the target and the model helps to approach the target. Perhaps discussions of induction have likewise been warmup exercises for more realistic engagement with a cultural issue of great import for intellectual history. Schurz is surely right to conclude that

a satisfying justification of induction would not only be of fundamental *epistemological *importance; it would also be of fundamental *cultural *importance as part of the enterprise of enhancing scientific rationality. (Schurz 2008, 280, emphasis in the original; see also Schurz 2019, 16)

Because everyone relies on induction most of the time, any real rival to science/induction would be ordinary induction qualified with *occasional* exceptions based on some alternative process—a kind of rivalry very rarely considered in the philosophical literature on induction, though computer science has paid more attention (Blum and Blum 1975; Minicozzi 1976; Case and Smith 1978; Angluin et al. 1983; Case and Smith 1983; Valiant 2013). Schurz and Thorn provide helpful terminology to describe a localized rivalry. “We also distinguish between prediction games with persistent and intermittent players. Persistent players deliver a prediction for each event, while intermittent players do not.” (Schurz and Thorn 2016) Clendinnen gives a rare philosophical example of an intermittent player:

If we consider a clairvoyant competing with science, it is clear that the clairvoyant can have a marginally higher success rate while using a method which does not reduce to induction. For instance, he normally accepts scientific predictions, but on two or three occasions makes different and conflicting predictions which turn out correct. If we consider a world with a very large number of clairvoyants whose predictions are mostly wrong, it is clear that there will be a method which succeeds more often than induction. This method will consist of normally trusting scientific predictions but on certain particular occasions trusting certain specified clairvoyants. (Clendinnen 1977, 106)

Meta-induction takes time to sort out any true clairvoyants (if there are such) from false ones. If this clairvoyant has merely been lucky, his or her success will not last and meta-induction will eventually expose him or her as unreliable if he or she keeps predicting. If the supposed clairvoyant has genuine real clairvoyant powers and keeps predicting, meta-induction eventually will notice that instead. Recent philosophical interest in robustness might inspire consideration of this kind of predictive method in a robust inductive logic. But what if the clairvoyant is genuine but dies or retires early? There is no guarantee that meta-induction will identify a genuine clairvoyant whose predictions are sufficiently few. Thus far, it seems evident that justifying induction is a difficult problem of considerable importance, and perhaps the most serious rival to (exceptionless) induction has rarely been considered in the philosophical literature. This threat has connections, however, to a recognized weakness in Reichenbach’s work. The rest of the paper will argue that meta-induction does not clearly justify (exceptionless) induction, but could be, and indeed has been, used to justify (part-time) prophecy. Much depends on what one does with testimonial evidence, which is less congenial to Schurz’s project than it perhaps seemed.

## Reichenbach’s Short Run Problem and Schurz’s Improvement

A major trouble spot for Reichenbach was the short run problem. He was aware of asymptotic rules that give the same limiting frequency as his preferred rule (Reichenbach 1938, 354–355; 1949, 447). But his answer was rejected even by Salmon (1955; 1963). The short run is more clearly relevant to our interests than the long run: if science finally starts outperforming fortune-telling in a million years, why trust science now (Friedman 1979)?

How does Schurz handle this problem? Because his meta-inductor infallibly keeps track of the success rates of all available predictors, the meta-inductor will stay not far behind the best predictor (roughly speaking), not only in the actual world, but in all possible worlds.

It must be emphasized that in demonstrating optimality one must allow *all *possible worlds, including all kinds of *paranormal *worlds in which perfectly successful future-tellers or anti-inductivistic demons do indeed exist. Restricting the set of worlds to ‘normal’ or uniform worlds would completely *destroy *the enterprise of justifying induction. For then we would have justify inductively that our real world is one of these ‘normal’ worlds, and we would end up in that kind of circle or infinite regress in which according to the Humean skeptic all attempts of justifying induction must end up. (Schurz 2008, 280–281, emphasis in the original)

Schurz goes to considerable effort to avoid begging the question against proponents of esoteric predictive methods, in order to craft an argument that ought to convince them that object-level induction is actually the best bet. His recent book is even clearer:

In particular, the justification of induction must not already presuppose a naturalistic worldview excluding the possibility of clairvoyance or spiritual connection with an omniscient God. All assumptions of this sort would make our enterprise circular because the justification of the naturalistic worldview presupposes our belief in the reliability of induction. (Schurz 2019, 80)

Thus Bradley’s presupposition of critical history, akin to Burks’ presupposition theory of induction cited above, will not suffice. Schurz, unlike Reichenbach, makes sure that the meta-inductor doesn’t fall far behind the best predictor(s) even at finite times—not merely focusing on a limiting frequency as Reichenbach did, but having nearly optimal success in the short run. This is a considerable improvement on Reichenbach’s short run problem. Rather than feeling free to design airplanes by consulting fortune-tellers, because maybe science won’t start working for a million years while fortune-telling racks up success after success in the meantime, we should, in light of Schurz’s argument, already be designing airplanes scientifically. (Recalling the benzene ring discovery, Ramanujan’s fairly reliable mathematical dreams featuring a Hindu goddess (Salmon 1984, 11), and Reichenbach’s distinction between the context of discovery and the context of justification, one should perhaps emphasize that the context of *justification* should be scientific rather than prophetic.) Fortunately, no one disagrees. But has the short-run problem been addressed so well as to exclude the kinds of rivals to induction that real people have actually embraced? I believe not, as will appear below.

One qualification is that if several evil demon predictors aim to fool the meta-inductor, life gets harder, as Schurz discusses (chapter 6). But this is a largely academic problem: evil demon skepticism is widely conceded to be both irrefutable and plausible to no one. Those who wish to try to solve radical skeptical problems are of course free to do so. But such an activity is radically different from, and much less urgent than, solving a skeptical problem with great cultural relevance, as Schurz commendably aims to do. I am inclined to accept Schurz’s claim that meta-induction is optimal as a response to Hume’s problem of induction. But the argument threatens to fall short just where it matters most.

## What about Historical Disagreement?

It turns out, however, that there is a largely unrecognized very-short-run problem for Schurz’s meta-inductive justification for induction, one that could potentially result in a meta-inductive justification of a (part-time) rival to induction.^2^ In this case, the short run problem pertains first of all to the past, and only derivatively to the future. A paper by Burks helps to set up the problem by calling attention to the difference between remembering the past and reconstructing it in light of a predictive method. Bradley, as we have seen, favors writing history by occasionally reconstructing the past in light of a predictive method when historical data contain elements incompatible with his inductive predictive method. Such views are dominant in intellectual circles today, strong even in theology (Langdon 1961; Harvey 1966) (though critics seem to be growing in influence (Wright 2003; Deines 2013; Bauckham 2017) and one can even find Bayesian methods (Heilig and Heilig 2016)), so Burks’ idealizations are not wholly without real-world application. Burks considers inductive (Star), random (Dagger) and inverse (Diamond) predictive methods.

In the first place, Mr. Dagger would challenge Mr. Star’s claims concerning the nature of the past. As Descartes and Hume pointed out, the character of the past is only known to a person at a given time via an inductive method starting from the data available at that time. Since Mr. Dagger uses a different inductive method to determine the nature of the past from that used by Mr. Star, he would arrive at a different conclusion concerning the past than Mr. Star did. Thus Mr. Dagger would not accept Mr. Star’s conclusion concerning the relative past successes of their respective methods. (Burks 1953, 187)

Each method, when used to *reconstruct/retrodict* the past, validates itself, just as judging a yardstick by itself shows it to be a yard long even if it is deformed. Retrodicting the past by a potentially non-inductive method is typically incompatible with remembering the past. If we remembered the past, we wouldn’t be trying to reconstruct it. A remembered past can be used to test methods, but a past reconstructed by some method cannot.

By contrast, Schurz’s meta-inductor *remembers the entire past*, so one would expect that Schurz’s results are realistic insofar as we all remember the past and agree on it. But most of the past is remembered by no one on Earth—not even the nineteenth century. Besides that temporal limitation to memory, there is also a spatial limitation because historical events have sufficient diversity and spatial volume that merely being alive at the time would not suffice to know about them all first-hand. We all rely on testimony due to both spatial and temporal limitations. How to do so responsibly has been the subject of much discussion from the philosophy of history to contemporary analytic epistemology and much else besides. A meta-inductive justification for induction must rely heavily on testimony as a form of surrogate memory.

If testimony were always reliable, then extending our memory(s) by testimony would be unproblematic. (Of course human memory isn’t perfectly reliable either.) Unfortunately, testimony isn’t always reliable. Given that some testimony favors the occurrence of counter-inductive events (miracles or the like), it is all the clearer that some testimony is unreliable—at least if (exceptionless) induction is justified. Hence justifying induction based on testimony, when a portion of the testimony seems to imply the occasional falsehood of induction, is a surprisingly delicate enterprise.

Given Schurz’s efforts thus far to avoid *assuming* the justification of induction and correspondingly to avoid begging the question against the esotericist, one is now surprised by what a leap is introduced. In the 2008 paper he writes:

But this analytic justification of *meta-induction *would at the same time yield an *a posteriori *justification of *object-induction *in the real world: for we know by experience that in the real world, noninductive prediction strategies have not been successful so far, hence it would be meta-inductively justified to favor object-inductivistic strategies. (Schurz 2008, 304, emphasis in the original)

And later he writes:

However, as we have explained in Section 2, this analytic justification of meta-induction implies an a posteriori justification of object-induction in our real word, because so far object-induction has turned out to be the most successful prediction strategy. This argument is *no longer circular*, *given *that we have a noncircular justification of meta-induction—and we have it.

The major advantage of the meta-inductivistic approach is its *radical openness *towards all kinds of possibilities. In my view, this radical openness is a sign of all *good *foundation-oriented (instead of ‘foundationalistic’) programs in epistemology. Unlike in Rescher’s “initial justification” of induction (1980, 82), meta-induction does not exclude esoteric worldviews or prediction methods from the *start*. Such an a priori exclusion would prevent a constructive dialog between a scientific philosopher and an esoteric-minded person. Meta-induction takes all these possible worldviews initially seriously and argues: wherever the ‘ultimate truth’ lies, you should in any case employ meta-induction because it is universally optimal among all accessible prediction methods. (Schurz 2008, 304 emphasis in the original)

I find both Schurz’s goals and most of his argumentation attractive and plausible, but I am puzzled by the abrupt introduction of the we-all-know clause about the track record of object-level induction after so much effort to avoid begging the question, postulation, *etc*., earlier. Doubtless most philosophers of science accept it, but what of the esotericists whom Schurz aimed to persuade? This premise turns toward the naturalistic choir to preach. Does the half or more of the world’s population that uses a hybrid method that sometimes contradicts induction really believe that its part-time esoteric predictive method(s) *have generally failed thus far*? If that were true, then one should find such people saying things like this: “Madam X’s crystal ball readings have been worse than chance thus far, but I think that she’ll start hitting the truth next week. I’ve made an appointment!” Track records would not be important for trying to predict future success rates. But no one is that foolish. Presumably proponents of esoteric predictive methods believe, reasonably or otherwise, that their esoteric means of prediction *have worked pretty well so far*, perhaps by outperforming induction in those rare cases of conflict, and/or by not conflicting with induction. Why else would they use such methods? Some form of rudimentary meta-induction is apparently widespread^3^. While philosophers of science mostly take ourselves know that induction has worked best thus far, the majority of the human race that uses part-time esoteric predictive methods presumably takes itself to know that one or another such method has either outperformed induction in cases of conflict, or is compatible with induction somehow. (How the Stoics held something like the latter view will appear below.) If there is a meta-inductive justification of induction, is there also a meta-inductive justification of fortune-telling, faith, or the like? Has it in fact been in routine use? Relativized to one’s beliefs about the past, it seems that there is. Recall Reichenbach for the logic:

we demand the forecast of the clairvoyant and compare it with later observations; if then there is a good correspondence between the forecasts and the observations, we shall infer, by induction, that the man’s prophecies will also be true in the future. (Reichenbach 1938, 354)

Were such a good correspondence to arise, one could speak of Reichenbach’s justification not of induction, but of induction-qualified-by-clairvoyance. This would be something of a disaster for the “scientific rationality” as desired by most philosophers of science and many scientists. Induction-qualified-by-clairvoyance would be no problem for designing vaccines and airplanes and proposing General Relativity and quantum mechanics, but it would undermine those claims, especially in historical sciences, where an *exceptionless* uniformity of nature is salient. Naturalistic science would be defeated intellectually, though knowledge, most of science, and all of technology would survive. Presumably clairvoyants and other proponents of part-time meta-inductive strategies would be quick to invoke Reichenbach’s argument form, if they knew about it. (Soon we will meet some ancient examples.) While Reichenbach seems not to have tried to check the success rate of clairvoyants (though some people were interested in such matters (Cerullo 1982), his envisaging only an example with a fairly disreputable label (“clairvoyant”) might suggest an underestimate of the sociological gravity of the problem. The fact that human memory covers so slight a field, necessitating testimony (and perhaps sifting thereof in light of some predictive method?), makes it easier to beg the question than Reichenbach seems to have expected. Even if my experience reveals induction to be the most successful predictive method along my world-line, should I ignore the reported experience of the rest of the human race if it differs?

There is a further difficulty nearby. Oftentimes (perhaps usually) where there is testimonial evidence in *favor* of the success of some esoteric predictive method, there is *no contrary testimony that inductive prediction worked better instead* (Vincent 1995, 14; Craig 1986). Thus it is not a matter of testimony for esotericism *vs*. testimony for induction, but rather testimony for esotericism *vs*. an *inferred absence* of data due to rejecting the data as unreliable. Where the esotericist offers a purported “memory” (testimony in the form of sacred scrolls, perhaps, or articles from the *Proceedings of the Society for Psychical Research*?), the inductivist offers only a blank, filled in by reconstruction by a predictive method (in accord with Bradley’s critical history). Thus Hume’s critique of miracles does not primarily counter testimony to miracles with other witnesses who were also present and report that no miracle occurred. (For sufficiently spatially or temporally widespread purported miracles, such as darkness at mid-day or relics that perpetually shed blood/milk/tears, contrary testimony might well be available.) Rather, the strategy is to cast doubt on the testimony that exists (filtering the evidence), and then fill in the resulting gap in the evidence by *reconstructing* the event à la Burks in accord with object-level induction, so the witnesses must have been dishonest, gullible, or the like. Such a strategy is expected from rationalists like Spinoza and Bradley, but is more curious in the hands of Hume the induction skeptic and supposed empiricist, as Broad observed (Broad 1916). If the Sun can fail to rise and bread can fail to be nourishing, why can’t dead people stop being dead, and why couldn’t there be evidence to that effect? That contrary testimony is often unavailable is no surprise, because reports of such events emerge especially among the faithful. As Hume puts it:

*Thirdly, *It forms a strong presumption against all supernatural and miraculous relations, that they are observed chiefly to abound among ignorant and barbarous nations; or if a civilized people has ever given admission to any of them, that people will be found to have received them from ignorant and barbarous ancestors, who transmitted them with that inviolable sanction and authority, which always attend received opinions. (Hume 2000, 90)

But aren’t the alleged events fulfilling the esoteric predictions simply implausible (or impossible)? Many rationalists have said so. Idealist F. H. Bradley was encountered above. Here is liberal theologian Rudolf Bultmann:

The historical method includes the presupposition that history is a unity in the sense of a closed continuum of effects in which individual events are connected by the succession of cause and effect […]. This closedness means that the continuum of historical happenings cannot be rent by the interference of supernatural, transcendent powers and that therefore there is no “miracle” in this sense of the word. (Bultmann 1960, 291–292)

Such events might well be implausible if one already knows that induction is justified, or if one is unaware of Hume’s problem (like many historians and Biblical scholars), or if one postulates a broadly *deistic* God who *would* not perform miracles and would prevent Humean alterations in the course of nature, yielding a coherent and self-recommending rationalistic liberal theistic belief-package, for example. Appealing to laws of nature, at least laws of nature with the typical content that would prevent dead people from rising and would keep bread nourishing and the Sun rising (or rather, the Earth turning), is not an admissible move, at least not unless one gives up taking the problem of induction seriously and renounces empiricism in favor of rationalism. But if one needs to justify claims that bread will remain nourishing and the Sun will continue to rise, as empiricists do, then one also needs to justify claims that dead people stay dead, *etc*., or whatever constitutes the failure of the esoteric predictions. If a dead person’s coming back to life is (for all we know) just one of those things that can happen from time to time, perhaps a Humean change in the course of nature (anything can follow anything) or the kind of event that spirits like to bring about once in a while, then it is not as easy to reject that testimony that a dead person did in fact rise. If one is prepared to invoke rationalism at *this* late stage to arrive at anti-supernaturalism, then one gives up the professed aim to persuade the esotericist masses and has poorly used one’s time as well. Why go to the trouble of a meta-inductive justification of induction, when one could have invoked rationalism to infer anti-supernaturalism right from the start like Bradley, Bultmann, *et al*.? It would seem that, thus far, meta-induction has offered a justification of induction that works where no one doubted it, but begs the question against perhaps the more serious alternatives or even threatens to justify some alternative instead (at least as esotericists might judge track records). No one doubts induction for most times and places (e.g., regarding Napoleon^4^), but what about the occasional purported exceptions where one or another esotericist claims a success?

The possibility of a predictive method that usually agrees with object-level induction but occasionally competes with it undermines the idea (Lee 2022) that one somehow has no choice but to accept induction if one has the goal of predicting the future. There are plenty of alternative ways to predict the future, which agree sufficiently with induction to justify science and daily life, while making rival predictions once in a while. As will appear, it isn’t obviously ruled out that these alternative predictive methods will even have some rational basis in terms of a good track record for the predictions that disagree with science. So, if coherence justifies something in the vicinity of induction, the justification is too coarse to distinguish exceptionless uniformity of nature from uniformity with a modest number of judiciously chosen exceptions.

## Meta-induction in Ancient Israel

Whereas the next section will recall the Stoic defense of divination and note its meta-inductive character, here it suffices to pick a few important and/or clear examples of the use of meta-induction in the Hebrew Bible to motivate apparently unreasonable (by ordinary standards) behaviors on religious grounds. Note that the question of historical reference, that is, whether these stories or anything like them actually happened, can be set aside for now. What *is* clear is that some ancient Hebrew writers, whether reliable historians, scheming priests, or anything in between, thought to make (with varying degrees of explicitness) their exemplary characters predict in accord with meta-induction as a justification for striking acts of faith in tension with inductive regularities, and their anti-exemplary characters predict in accord with object-level induction on meta-inductively contraindicated occasions. These passages show surprising philosophical sophistication and yet expect their readers (and potentially non-literate hearers) to understand and accept the implied argument due to mere invocation of the track record. Perhaps meta-induction is an innate form of reasoning?

Abraham’s life is a good place to find meta-inductive prediction. Judaism, Christianity, and Islam all claim to be derived to some degree from Abraham. One of his most striking acts of faith (according to Genesis) is his obeying God’s command to sacrifice his son Isaac^5^—the son whom God had promised through Abraham’s (postmenopausal) wife Sarah and through whom his theologically important descendants were to come—at God’s command (Genesis 22). This sacrifice seems imprudent in the extreme if Abraham was hoping to have many descendants through Isaac, as God had promised earlier. But given that Isaac’s mother Sarah was postmenopausal when he was conceived (Genesis 18:11–12), Isaac’s very existence was a striking case of prophecy’s outperforming inductive gynecology. With religion having strikingly defeated science (that postmenopausal women do not bear children) at least once in Abraham’s life, he presumably reasoned meta-inductively that God’s promises would be more reliable than science (that slain sons stay dead and, in any case, do not produce grandchildren) again. Hence the promise of descendants indicated that Isaac would somehow survive the process of Abraham’s undertaking to sacrifice him. Mercifully, the sacrifice is stayed at the last minute by a new message from God, who was perhaps satisfied that Abraham had rejected object-level induction in favor of meta-inductive faith in God’s promises.

A second formative event in the nation of Israel, according to the Pentateuch (in this case Exodus through Deuteronomy), was the inheritance/conquest of the land of Canaan. God had promised the land to Abraham and his descendants, but they were enslaved in Egypt. Moses and God got the Israelites out of Egyptian slavery *via* miraculous plagues, fed and watered the Israelites in a wilderness for a long time, and then expected the Israelites to believe God’s promise of inheriting the land of Canaan. Canaan, however, was by no means empty, but was fortified with walled cities and inhabited by people with high-tech weapons (iron chariots) and/or considerable size, people who were not planning to move out or fall dead before the Israelites. The ethics of the conquest of Canaan is a perennial topic for Jews and Christians, though it did not seem to bother the Israelites all that much at the time. The inductive logician, however, should be especially interested in the story from the standpoint of military science. It is often emphasized how unpromising a military position Israel had in attempting to conquer the fortified cities of the promised land of Canaan, given the obstacles mentioned above, not to mention presumably few or poor weapons in the Israelites’ hands and the presence of noncombatants, livestock and worldly goods in their nomadic condition. However, success was predicted meta-inductively by Moses on the grounds of God’s having rescued the Israelites from slavery in Egypt *via* the plagues and parting the sea. The great bulk of the Israelites, however, still reasoned in accord with object-level inductive military science, concluding that they could not conquer Canaan. God, however, is a meta-inductivist, as shown when expressing irritation that the Israelites predicted military outcomes using object-level induction rather than meta-induction.

And the Lord said to Moses, “How long will this people despise me? And how long will they not believe in me, in spite of all the signs which I have wrought among them?” (Numbers 14:11 RSV)

God and Moses had an impressive track record in the Egyptian plagues and the wilderness, in that the Israelites were not defeated by Egypt’s army (contrary to military science), were not drowned in the sea (contrary to hydrodynamics, though Egypt’s army was), and did not die of hunger or thirst in the wilderness (contrary to physiology and agricultural science). So the Israelites, we are told, should have expected God to enable them to conquer Canaan. Far from being a highly theoretical form of reasoning requiring explicit instruction, meta-induction was apparently too obvious to require explanation to common ancient Near Eastern peoples; it was only necessary to call attention to the track record and infer the conclusion. The punishment for object-level inductive rather than meta-inductive reasoning was to wander in the desert most of 40 years until all the military age men had died—except the anti-Moses ringleaders, who were struck dead by a plague, and the handful of the faithful. Meta-induction was not just a good idea, it was the law in ancient Israel, according to the Pentateuch.

A third example comes from the later historical books. According to the Law of Moses (the Pentateuch), Israel should not make foreign alliances or fear foreign armies, because God promised to defend them. After united Israel had split into northern Israel and little Judah at the south, Judah’s King Asa removed paganism (II Chronicles 14). Judah was attacked by Zerah the Cushite with a vast army. Asa prayed, and the Lord struck down the Cushites before Judah. Later Asa, in less desperate circumstances, chose a more worldly, inductive path (II Chronicles 16): he sent treasure from his palace and from the Temple to Aram (Syria) to break Aram’s treaty with Israel, Judah’s assailant. It worked, but the prophet Hanani was not impressed:

“Because you relied on the king of Aram and not on the Lord your God, the army of the king of Aram has escaped from your hand. Were not the Cushites and Libyans a mighty army [...]? Yet when you relied on the Lord, he delivered them into your hand [...]. You have done a foolish thing, and from now on you will be at war.”

While the prophet Hanani (or whoever wrote this story, as the case may be), like God in the book of Numbers, has not gotten much ink in the history of inductive logic, he clearly reasoned meta-inductively and expected Asa to have done the same. I would not say that the ancient Hebrews invented the pragmatic justification of induction—it seems that they had no problem of induction, and it is by no means clear that they accepted the resulting religious claims merely pragmatically (i.e., with no claim that the religious predictions were true or probably true, but merely the best available), but they showed clear instances of meta-induction. Depending on one’s upbringing, one might be able to produce additional examples, but these should suffice for present purposes. Whether one takes these stories to have actually happened is of course another issue, though (much as Schurz urged above) one should resist the temptation to beg the question by rejecting them due simply to their non-natural character, if induction needs to be justified. But it is already interesting that seemingly irrational behavior in some key events portrayed in Hebrew Biblical history has an implicit or partly explicit meta-inductive basis. Somebody(s), whether Moses or not, whether writing history, fraud, or fiction, reasoned meta-inductively in writing the so-called Books of Moses, *etc*. Perhaps “fundamentalistic” reasoning is less common and meta-induction is more common in religious thought than Schurz has suspected? While meta-inductive reasoning can be found in the Hebrew tradition, it became more explicit among Greek philosophers in connection with divination at Delphi and other oracles.

If one goes about believing and doing risky anti-inductive things like the Biblical heroes do, clearly there is a premium on not fabricating or misunderstanding occasions for non-inductive predictions. Presumptuously trusting ‘promises’ that God hasn’t made, such as that Solomon’s Temple in Jerusalem would never be destroyed (as in Jeremiah’s day), does not work out well. Picking fights with science tends to be a bad idea.

## King Croesus and the Stoic Defense of Divination *via* Meta-induction

Meta-induction as a logic of prophecy—not merely as an idle threat as for Reichenbach, but as a case taken seriously by real human beings, some of them outstanding philosophers such as Chrysippus and Posidonius—becomes explicit with the Stoic defense of divination. Unfortunately, much of that Greek material is lost, but we do have a summary in Cicero’s *On Divination*. Before meeting the Stoics *via* Cicero, let first us encounter King Croesus’ experimental parapsychology, which serves as a clear example.

Herodotus recounts how the proverbially wealthy king interacted with the Greek oracles in what one can only regard as a meta-inductive fashion:

he at once made inquiries of the Greek and Libyan oracles, sending messengers separately to Delphi, to Abae in Phocia, and to Dodona, while others were despatched to Amphiaraus and Trophonius, and others to Branchidae in the Milesian country. These are the Greek oracles to which Croesus sent for divination: and he told others to go inquire of Ammon in Libya. His intent in sending was to test the knowledge of the oracles, so that, if they were found to know the truth, he might send again and ask if he should undertake an expedition against the Persians. (Herodotus 1920, Book I, ch. 46, sect. 1)

The test involved having messengers, 100 days after leaving Croesus, ask the oracles what Croesus was then doing. While some of the oracles evidently failed the test, the Pythian priestess at Delphi had this to say:

“The smell has come to my senses of a strong-shelled tortoise

Boiling in a cauldron together with a lamb’s flesh,

Under which is bronze and over which is bronze.” (Herodotus 1920, Book I, ch. 47)

Evidently she was right:

when he read the Delphian message, he acknowledged it with worship and welcome, considering Delphi as the only true place of divination, because it had discovered what he himself had done. For after sending his envoys to the oracles, he had thought up something which no conjecture could discover, and carried it out on the appointed day: namely, he had cut up a tortoise and a lamb, and then boiled them in a cauldron of bronze covered with a lid of the same.

H. H. Price dubbed Croesus the unacknowledged founder of psychical research (Price 1940): “Croesus argued, reasonably enough, that if the Delphic priestess had clairvoyant powers, she probably also had the precognitive powers which she claimed to have.” I point out that Croesus’ reasoning is meta-inductive; perhaps Croesus also deserves a place in the annals of inductive logic. He tested the oracles to see if they had superhuman knowledge in one clear instance, and then meta-inductively expected superhuman knowledge in further instances. The successful track record is short but not trivial. (Croesus later found the oracle’s guidance in relation to Persia tragically ambiguous.)

Divination was defended in detail by Stoics including Chrysippus and Posidonius (though not much is left to us) and in Latin in the first book of Cicero’s *On Divination* (Cicero 1923), in which the character Quintus (Cicero’s brother) defends divination before Cicero ‘himself’ (Marcus Tullius) attacks it in the second part. It is no longer widely assumed that Cicero’s own philosophical voice is that of the character with his name (Fox 2007, 214). It is observed that Cicero was himself an augur (Roman state diviner), that the two parts of the dialog should be read in balance especially in light of the precedent of presenting two sides without passing judgment (Beard 1986), and that Quintus’ arguments are not effectively refuted in the second part of the work (Schofield 1986; Denyer 1985). Cicero’s work has recently received attention in the philosophy of science (Cabrera 2020). Of greater significance than Cicero’s own view, however, is that important Stoics had defended divination with arguments of the sort provided by Cicero. He distinguishes between natural divination (messages from a god *via* a prophet(ess)) and technical divination (observing animal entrails, directions of bird flight or pecking behavior or other intrinsically meaningless events held to be correlated with future events).

Quintus says the following, which we can take as a sketch of the Stoic defenses that seem largely lost to history:

Speaking now of natural divination, everybody knows the oracular responses which the Pythian Apollo gave to Croesus, to the Athenians, Spartans, Tegeans, Argives, and Corinthians. Chrysippus has collected a vast number of these responses, attested in every instance by abundant proof […]. But I pass them by as you know them well. I will urge only this much, however, in defence: the oracle at Delphi never would have been so much frequented; so famous, and so crowded with offerings from peoples and kings of every land, if all ages had not tested the truth of its prophecies. For a long time now that has not been the case. Therefore, as at present its glory has waned because it is no longer noted for the truth of its prophecies, so formerly it would not have enjoyed so exalted a reputation if it had not been trustworthy in the highest degree […]. [What] cannot be denied without distorting the entire record of history, [is] that the oracle at Delphi made true prophecies for many hundreds of years. (Tullius Cicero 1923, 267–269)

This is a meta-inductive argument for trusting a form of prophecy. Because of true prophecies made in the past at Delphi (which people recognized gratefully by giving lavish gifts), one should expect true prophecies from there in the future. At least one should have until Delphi’s powers started to wane, which the track record also showed.

For the Stoics, a meta-inductive argument for divination was not in competition with a meta-inductive argument for science, because science and divination had non-overlapping domains. In fact, the Stoics favored both arguments and saw the two as compatible (Sambursky 1959). That makes sense when divinatory predictions involve natural (as opposed to supernatural) events. “Basing themselves on their strictly deterministic doctrine, the Stoics denied any essential difference in method between scientific inference and inductive divination” (1959, 67), although scientific prediction is intelligible and divination is not, Sambursky says.

[T]he postulation of determinism (i.e. fate) implies the assumption of the validity of the method of induction (i.e. divination).

[…] On the other hand, the fact that divination “worked”, and that the diviners had their successes, was a strong argument in favour of causality. Each prediction that came true added a new instance to the sum total of all the cases noted by experience where the event *B* followed every time that the factual situation *A*, as seen by the diviner, repeated itself. Thus for the Stoics the ever-lengthening chain of fulfilled predictions increased the belief in future successes and represented an experimental proof for determinism based on the generalization of the observed concatenation of the events *A* and *B*. (Sambursky 1959, 68–69)

The extent of the successful track record of divination, however, was controversial, though proponents admitted failures and critics admitted successes (1959, 69). According to Hankinson, the case for divination was akin to a method used by Empiricist physicians (Hankinson 1988), which is elsewhere likened to “the attempts of some to produce self-supporting arguments for induction as a valid procedure in science” (Hankinson 1987). This last remark perhaps most closely resembles inductive defenses of induction along the lines of Max Black, but also resembles the meta-inductive streak in Reichenbach’s work. Schurz’s emphasis on the meta-inductive side of Reichenbach’s pragmatic justification gives an even closer fit with the virtue of non-circularity.

Broadly similar things might be said about the Jewish tradition (which also underlies Christian and Islamic belief at some level), but also with key differences. Like Greek divination, the Hebrew tradition also contains both (narrowly prescribed) technical means of learning God’s will (e.g., the *urim* and *thummim *and/or an ephod) and an oral/written message from God or a prophet, hence versions of technical and natural divination, respectively. But the Hebrew tradition contains crucial predictions that are physically impossible, such as the birth of Isaac to post-menopausal Sarah. The fulfillment of such events obviously would not strengthen a case for *physical *determinism. Thus, in the Jewish tradition sometimes one might have to choose between scientific and prophetic predictions regarding, say, whether Sarah will bear a child. Thus, uniformity-based science and religion could conflict occasionally, but, we are told, experience shows that religion won. This claim suggests that the usual science-religion warfare thesis, according to which science wins, might also be also meta-inductive.

Given that meta-induction not only can be invoked as a logic of prophecy, but already was by the Stoics, and implicitly by the Israelites as well, it is too quick merely to assume or assert without investigation that experience vindicates science full-time (exceptionless induction) and never vindicates prophecy. One could envisage as alternatives, a Stoic view in which science and divination coexist peacefully (not competing because the divined events are not obviously supernatural and because divination applies in realms to which no science applies (Denyer 1985) or a Jewish view in which science and prophecy occasionally conflict but prophecy wins, for example, views already available in the ancient world. It would be interesting to know why such ancient and familiar material has not been connected more often with inductive logic. Probably the dominance of deduction in philosophy for two millennia, roughly what Grünbaum and Salmon called deductive chauvinism (Adolf and Salmon 1988), played a key role. The connection to Apollo and Zeus cannot have helped.

## A Logical Empiricist Justification of Faith?

The logical empiricists’ obvious naturalistic and anti-metaphysical inclinations and Carnap’s supposedly claiming that theology is meaningless (Carnap 1931) would seem to make them perhaps the least likely people to work out any kind of logic of traditional religious faith—a logic that, naturally, requires empirical premises. The surprise lessens somewhat if one recalls how early modern rationalism often competed with orthodox faith, or how orthodox Aristotelian philosophy (Averroism) often competed with all the Abrahamic faiths in medieval times (such as regarding the eternity of the world *vs*. creation in the finite past), making empiricism and philosophical skepticism potentially attractive to the religious. William James is a relatively recent proponent of empiricism as pro-religious:

Let empiricism once become associated with religion, as hitherto, through some strange misunderstanding, it has been associated with irreligion, and I believe that a new era of religion as well as of philosophy will be ready to begin. (James 1912, xiii)

Even the young Carnap’s denunciation of metaphysics makes clear that theology that makes empirical claims is meaningful, though then “subject to the judgment of empirical science.” While Carnap presumably thought this was an unpromising situation for traditional theologians, only liberal theology, the kind that makes no empirical claims, was meaningless for Carnap (1931, 66–67). Someone sufficiently traditionalist to claim empirical content for some theological claims will not find so much tension between logical empiricism and the meaningfulness of theology, even granting the verificationist criterion of meaning (Price 1935).

It may have been left, ironically, to the logical empiricists to combine these two ingredients (skepticism and meta-induction) and thereby unintentionally clarify the logic of Abrahamic faith (insofar as one exists): empiricist induction skepticism undermines a priori rationalist critiques of miracles, while meta-induction, in largely filling the hole left by empiricism, potentially includes prophecies with good track records and/or miraculous events. The idea that skeptical philosophy is congenial to Abrahamic religion has a long history studied especially by Richard Popkin, historian of skepticism *par excellence*, throughout his career (Popkin 1956; 2003). Popkin, who as an adult came to take his Jewish heritage with religious seriousness, embraced this view himself (Popkin 2003, xxiii). The Stoic defense of divination, though of course ancient, seems to have had few admirers for a long time, perhaps partly because its argument* form* was not so easily detached from its Greek pagan roots involving prophecies ascribed to Apollo or Zeus. Given the exclusivism of Abrahamic religions, since *c*. 500 A.D. most thinkers will have wanted to conclude that the Greek oracles at Delphi and elsewhere were either demonic or uninspired (false, ambiguous, trivial, lucky, *etc*.); clearly the latter is more congenial to Enlightenment views. Any striking successes could, if necessary, be attributed to Satan *et al.*, given the common Christian view that behind the pagan gods were only demons (Patrides 1965; I Corinthians 10:20). But cleansing the Stoic argument for prophecy, powered (insofar as it was successful) by demonic activity associated with pagan *cults*, must have been more challenging than, say, cleansing teleological arguments to which pagans might affix the name of Zeus or the like. Either way—whether oracles were phony or demonic, or some combination of the two—there was a *prima facie* disincentive to find an interesting contribution to inductive logic, especially one congenial to Abrahamic faith. But contemporary philosophers should not be so easily deterred. Hence both skepticism as an aid to faith and meta-induction as a defense of prophecy are old ideas held by some religious philosophers to be pro-religious (even if the religion in some cases involved Apollo and Zeus). Instances of skepticism and meta-induction being held *together*, however, do not come easily to mind (at least not to my mind) in the history of philosophy prior to Reichenbach.

Even the most enthusiastic proponent of finding in meta-induction a logic of faith, however, should admit that there is little more than a sketch, and that filling in specific details so as to get detailed results, such as that one should accept doctrine *X*, would face enormous difficulties. What, for example, counts as the same predictive method over time? In formal epistemology this issue is settled by stipulation or computer programming, but it is much less clear in the real world, not least because humans die, though some are supposedly mouthpieces for the eternal. This question is related to traditional questions about identifying true prophets, ascertaining the inspired canon, or some such, a difficult task not made much easier by meta-induction. What then, if anything, would be achieved by identifying meta-induction as a partial logic of faith? Perhaps two things would result. First, one would have an argument potentially displacing rationalistic critiques of miracles by providing an alternate empiricist standard of rationality according to which the occurrence of such events and their acceptance based on testimony is not always clearly absurd and might even have positive epistemic status. Second, one would have a suggestion that faith is not inherently irrational or (with the later Wittgenstein) arational. Instead, some paradigm faith events that seem unreasonable might make sense meta-inductively. Those unsympathetic to faith might be glad to undermine the attitude (perhaps suggested by Tertullian or Kierkegaard) of a fideistic embrace of absurdity in favor of argument forms redolent of logical empiricism, thus reducing disagreement.

## How Is Anthropology Affected?

The question whether stories of supernatural events count as evidence has recently become contentious among American anthropologists, with legal implications for academic freedom for science. In this case orally transmitted legends are being used to decide whether scientific evidence can be gathered or retained in some cases in America. At issue in part is whether bones studied by anthropologists belong to the ancestors of specific Native American tribes, who would then have a strong claim to have the bones repatriated for reburial. The Native American Graves Protection and Repatriation Act, at least as employed in practice, arguably counts Native American religious oral traditions as dispositive historical evidence in some cases, in line with postcolonial thought currents but in tension with what one usually expects of scientific anthropology and of American jurisprudence regarding religion-state relations, argue physical anthropologist Elizabeth Weiss and co-author James Springer (Weiss and Springer 2020). Given the radical enhancement of postcolonial thought currents in western societies and even in recent American and British academic hiring patterns and university policies in the wake of George Floyd’s death in the custody of Minneapolis police, this kind of issue is likely to grow in importance. It would be interesting to know whether meta-inductive reasoning appears in these Native American traditions. In any case it seems evident that anthropologists’ need to address foundational questions in their discipline might make the philosophy of science relevant once again (Merrilee Salmon’s work being an earlier example (Salmon 1982). The Weiss & Springer book has induced a backlash, including moves by leaders at San José State University, Weiss’ employer. She has filed suit, claiming retaliation that limits access to the materials needed for her research, violating her academic freedom (Flaherty 2022). On the other hand, one can also sympathize with the Native American position on ethical grounds.

## Schurz on Testimony

Much as Reichenbach fleetingly entertained the possibility that clairvoyance could be vindicated empirically *via* a meta-inductive argument, Schurz admits that testimonies could *in principle* vindicate religious views: in certain (non-actual) circumstances “open-minded scientists would have to accept them.” (Schurz 2019, 210) In contrast to much of his earlier work on meta-induction, Schurz has felt the need in his recent book to address this worry about a possible testimonial basis for esoteric predictive methods. In a passage that does not easily fit the preceding 15-odd years of his work on the topic, Schurz allows that esoteric predictive methods are “sometimes” “fundamentalistic” (which for Schurz means blind trust in authority) but “often” based on “alternative” testimony, such as to miracles (2019, 210, also 43).

What makes this testimony “alternative” is unclear, given that frequently (perhaps usually) testimony to miracles is not contradicted by induction-friendly testimony, as noted above (Vincent 1995, 14; Craig 1986). If testimony to a miracle is the only testimony that we have regarding what happened at a particular place and time, in what sense is it alternative testimony? Does Schurz believe that induction is justified a priori after all (rejecting empiricism in favor of rationalism), hence justifying strict scrutiny for testimony to miracles? Perhaps alternative testimony is analogous to alternative medicine?

For reasons that are not clear to me, Schurz portrays such inferences as involving ordinary objection-induction from such alternative testimonies. It seems to me that it is actually often *meta-induction* from what the esotericist takes to be a record of successful predictions that is held to justify trusting the esoteric information source in the future. Perhaps uncritical believers do not ask why they should accept their esoteric sources. But as we have seen, some key characters in the Hebrew Bible, not necessarily culturally elite, *do* answer such questions in terms of track records of predictive success, perhaps because of the risky actions that they contemplated.

In sections 8.1 and 10.2 Schurz offers guidance on when testimony is acceptable. Rather than granting testimony the benefit of the doubt in contexts with large error rates and wishful thinking (such as forecasting or healing methods), one should require that the acceptance of beliefs through testimony be disciplined by attending to track records of success (Schurz 2019, 296). Thus far this is a step that everyone should welcome, I think. But Schurz’s introduction of criteria for the acceptance of testimony is a crucial supplement to what previously seemed intended as a largely complete defense of object-level induction *via* meta-induction (Schurz 2008). His criteria are also rather stringent, almost seeming designed to exclude the kinds of events around which miraculous testimony often comes, especially in relation to the remote past—and what events do not eventually become the remote past? “None of the religious testimonies known to me satisfies these criteria” (Schurz 2019, 210) of reproducibility and the presence of many independent witnesses, he reports. The reader is left somewhat unclear about which testimonies are known to Schurz, however; the book’s bibliography shows one religion-friendly source (an article by Oliver Crisp), one relevant hostile source (an article by Rob Pennock), and a great many works that do not obviously or systematically address the topic. The testimony surrounding St. Joseph of Cupertino’s numerous reported levitations might come closer than most other candidates to meeting Schurz’s criteria—at least for people in seventeenth century Italy. For that matter, Jesus is reported to have done many miracles, some in front of large audiences, on diverse occasions, while refusing to work miracles on demand from hostile parties. Were Schurz’s criteria satisfied for people at least in those times and places? Perhaps they were; the criteria are not so clear. Are Schurz’s criteria satisfied for people today? Most likely not; what it would it be for, say, resurrections to be repeatable? Must Jesus die and then rise from the dead every Easter? Must someone rise from the dead every Sunday in each diocese (one hopes not the same person from week to week)? If there are important one-time miraculous events in history, for which repetition is inappropriate, Schurz cannot come to know about them. If the position were merely one of great caution, motivated by fear of being wrong in a certain way, then one could suspend judgment about such testimony, rather than rejecting it. In any case the criteria seem more plausible as indicating when one *should* or *must* accept testimony, not when one may do so—unless one knows in advance that miracles are improbable, a claim hardly available when the justification of induction is in doubt, or one knows that any being(s) capable of performing miracles should or would perform them to suit the epistemic convenience of detached or hostile parties. Rationalism seems to be entering through the back door. If that is to be avoided, then a role for judgment seems to emerge once more, perhaps recalling Howson’s subjective prior probabilities. Suffice it to say that Schurz’s recently introduced criteria for testimony play a crucial role in his book’s defense of object-level induction *via* meta-induction, and that those criteria are not obviously of a piece with his case for meta-induction.

One might also compare Schurz’s views about the testimony for miracles with the views of a recent historian, John Vincent.

History is about evidence, but only about evidence we approve or. Evidence we disapprove of, might as well not exist. We decide, even before looking at it, what can be evidence and what not. Thus with miracles; thus with witchcraft.

The body of evidence in favour of miracles could not be of higher quality. It is based on the testimony of eye-witnesses. It is contemporaneous. It is massive in bulk. It comes from educated men. It is often entirely disinterested. It varies little over a large number of centuries. There is little evidence contrary to the idea of special providences, and perhaps there could not be. The historical evidence for miraculous intervention could not be weightier. Our decision to disregard it is not a historical decision, not an induction from the evidence as it is. It is based on our non-historical or a priori belief (for belief it is) that there is no such thing as the miraculous.

The same with witchcraft [...]. The evidence for witchcraft is in some ways of even higher standing than that for miracles. For it is sworn evidence, evidence given in court, fully and instantaneously recorded, a matter of due process of law, evidence very widely accepted not just by popular opinion but by the official mind. It is thus evidence, however wrongly obtained or subject to ulterior motive, which does not differ in form from that given in other legal cases of the period.

If we disbelieve in witchcraft, it is despite the evidence. History is about evidence, but we decide, or the general culture of our day decides, what to exclude from evidence. (Vincent 1995, 13–14)

(Praising the evidence for witchcraft, presumably as a tacit criticism of empiricism, has precedents (Lecky 1884)). Whereas Schurz is in principle open-minded toward evidence for miracles, his criteria are well designed to exclude isolated irregular occurrences from satisfying those criteria. Vincent, on the other hand, evidently finds the testimony for miracles (and their evil twin, witchcraft, which even defenders of miracles do not want to accept) very impressive, without imposing criteria about timing and audience that exclude the evidence as we find it, but simply rejecting the evidence because it is for miracles. What Vincent would make of the problem of induction, which historians do not face, but which makes it more difficult to exclude miracles in advance, is difficult to say.

## A Way Forward?

Perhaps one can concede that the successfully predicted events claimed by esotericists are not so implausible in themselves when the justification of induction is in doubt, but still are implausible all things considered because such events ought to have *other observable consequences*, *which are in fact not observed*. Hence the esotericist’s claimed successful track record, no longer confined to the sacred scrolls, is undermined. Such an approach has been suggested by Schurz in correspondence and in his book (Schurz 2019, 208). This is a promising strategy. Is it disappointing that an apparently clean argument (akin to the silver bullet that Earman finds Hume to have sought against miracles (Earman 2000, 4)) for exceptionless object-level induction requires poring over old texts and digging up rocks and fossils or pottery and then making judgments after all? Perhaps not: Schurz, in claiming that we all know that induction has worked best thus far, perhaps was *presupposing* some widely received views about the results of such inquiries as they occurred in the modern West, perhaps the destructive “higher” Biblical criticism, perhaps the outlines of geology (Schurz 2019, 208), perhaps archaeological problems finding traces of a multitude of Israelites camped in a desert for four decades (Finkelstein and Silberman 2001), or the like.

On the other hand, those inquiries were generally carried out without recognizing Hume’s problem of induction. The determined esotericist, one who does not easily embrace question-begging arguments against esotericism, might notice the Duhem-Quine problem of the need for auxiliary hypotheses in deriving those other missing empirical consequences. Are unrecorded secondary miracles (falsifying the auxiliary hypotheses) needed either to make the recorded success stories possible at all or to explain the missing traces of those success stories? If so, are such posits intrinsically implausible, or in contradiction to the basic thrust of the esotericist’s worldview (perhaps divine veracity or goodness), or likely to lead to a self-undermining skepticism? One should also not overlook confirmation bias, which seems likely to play a role in generating an exaggerated list of successes and an understated list of failures for esoteric prediction.

Philosophers of science apparently don’t generally find testimonial evidence for miracles very threatening. One reason why, following Hume on miracles, might be that esotericism is not a united front. There could be, and likely have been, rival esoteric predictions on some occasions. Hence the multiplicity of esoteric predictors could be a weakness. Moreover, some have claimed confirmation of doctrines by miracles. Mainline Protestants traditionally liked to claim that miracles confirmed doctrines and hence that purported miracles that occurred in the context of false doctrines (e.g., distinctively Roman Catholic) were illusory—so somehow the testimony was unreliable. This was a substantially arbitrary game that deists later played to reject *all* miracles (Brown 1984). (This Protestant claim is quite distinct from the claim, more typical of Roman Catholics, that miracles associated with false doctrines were demonic, leaving the testimony to miracles intact while making their doctrinal import ambiguous.) But there are ‘too many’ miracle claims, associated with incompatible doctrines, to accept them all, according to Hume’s contrary miracles argument:

let us consider, that, in matters of religion whatever is different is contrary; and that it is impossible that the religions of ancient ROME, of TURKEY, of SIAM, and of CHINA should, all of them, be established on any solid foundation. Every miracle, therefore, pretended to have been wrought in any of these religions (and all of them abound in miracles), as its direct scope is to establish the particular system to which it is attributed; so has it the same force, though more indirectly, to overthrow every other system. In destroying a rival system, it likewise destroys the credit of those miracles, on which that system was established; so that all the prodigies of different religions are to be regarded as contrary facts, and the evidences of these prodigies, whether weak or strong, as opposite to each other. (Hume 2000, 91)

Clearly strong background assumptions about the abilities, character and goals of any beings able to violate laws of nature were assumed by Hume, assumptions that happen to match those of mainstream eighteenth century Protestantism. Earman gives a Bayesian analysis:

In sum, Hume’s contrary miracles argument has some effect against those who take miracles to be proofs of religious doctrines. But against those who take miracles only as providing confirmation of religious doctrines, Hume’s argument is not vouchsafed by any valid principles of confirmation—at least not of the Bayesian variety. Hume is thus forced to leave the high ground and descend into the trenches. (Earman 2000, 70)

A similar fate seems to befall the meta-inductive justification of induction in its present form (apart from Schurz’s criteria regarding testimony). The argument was apparently not supposed to require any prior plausibility judgments of world-views, judgments of plausible approaches to the Duhem-Quine problem, detailed examination of old texts, digging up rocks and fossils, digging up pottery, *etc*. (Schurz 2008). Schurz’s meta-inductor infallibly witnesses and remembers everything. Consequently, the meta-inductor has no need for discernment, careful study, synthesizing a variety of fields, weighing the judgment of others in other fields, *etc*. But how do the meta-inductor’s conclusions immediately help *us*, who conspicuously lack such perfections and so must make such judgments time and again? While such questions are interesting, and might be answered in such a way as to vindicate object-level induction, one notices that they involve considerations that go well beyond the initial setup of the meta-inductive justification of induction. Howson’s view that Hume’s problem is insoluble except insofar as one feeds in opinions *via* prior probabilities (Howson 2000) seems to reemerge.

The need to attend to priors is in some respects no surprise. It is a familiar point that one can devise theories that fit the data really well if one allows them to be contrived, *ad hoc*, unnatural, or the like (Kukla 1996). Can one therefore hope for wave after wave of facticity to ensure the convergence of opinion in the long run—recalling that Bayesian convergence of opinion theorems can fail if theories are underdetermined by evidence (Earman 1992, 151)? It would seem that one could contrive an esoteric view that purports to enjoy the full collection of successful predictions recorded in its sacred scrolls and the full collection of successful predictions of modern science as well. As is well known, a view of that sort was presented in 1857 by naturalist P. H. Gosse, aiming to do full justice to the Bible and to geology (Gosse 1998). According to Gosse, God created fossils directly. Such a view generally was not and apparently is not considered optimal even by most believers in the Bible, however. (It also omits Noah’s flood, which makes the exercise fail on its own terms, a point rarely noted.) Why not? While most feel the implausibility of such a view, articulating a philosophical argument (as opposed to a gut feeling, a metaphysical postulate, or a theological argument) is not easy (Earman 1977; Norton 2011; Manchak 2011). Hence the widely shared aversion to such views suggests a need for something that the current meta-inductive argument lacks, namely, plausibility judgments, such as Howson’s priors. The resulting reasoning also bears some resemblance to Goodman’s picture of induction:

The point is that rules and particular inferences alike are justified by being brought into agreement with each other. *A rule is amended if it yields an inference we are unwilling to accept; an inference is rejected if it violates a rule we are unwilling to amend*. The process of justification is the delicate one of making mutual adjustments between rules and accepted inferences; and in the agreement achieved lies the only justification needed for either.

All this applies equally well to induction. An inductive inference, too, is justified by conformity to general rules, and a general rule by conformity to accepted inductive inferences. Predictions are justified if they conform to valid canons of induction; and the canons are valid if they accurately codify accepted inductive practice. (Goodman 1983, 64, emphasis in the original)

Of course there is no guarantee that “we” all arrive at the same reflective equilibrium, much as is the case with Howson’s subjective Bayesianism. If one clings to pure facts and renounces opinions, then one encounters much the same problem that befalls likelihoodism, namely, that there are interesting and meaningful questions that have no answers, such as how probable theories are in light of the evidence (Royall 1997; Sober 2008; Pitts 2013)—a question that one would surely have preferred to answer if possible. But one shouldn’t overstate the problem. Obviously one should milk the facts for all that they are worth. In the context of meta-induction, there will presumably be many esoteric views with a poor track record. There might be new meta-inductive simulations to run, perhaps with some uncertain observations, missing memories, or the like, that could shed light on such issues. Such work might give part of the answer to Hume’s problem, but there will be some subjective remainder requiring at least implicit attention to priors. Philosophy could become boring if fundamental questions often received final answers. Fortunately boredom should not set in here, because there is more work to do.

## Footnotes

1 The word “revelation” need not construed as a theologically important doctrine; successful predictions might provide grounds for expecting more successful predictions, without addressing any doctrines.

2 Schurz has felt the need to address this objection briefly in his 2019 book, on which more below, perhaps due to my influence from correspondence, conference talks, and/or other means.

3 One finds track record-based justifications of predictive powers in such diverse environments as financial advisors' newsletters (X predicted such-and-such investments that paid off well, so plausibly X's future predictions will be profitable also, one is invited to infer) and popular movies including *Live Die Repeat:* *Edge of Tomorrow* (where Cruise's character, having repeatedly experienced the future, knows what is coming and convinces various others to that effect) and *Imagine That* (where Eddie Murphy's character gives good investment advice due to his daughter's magic blanket). This type of reasoning is generally understood without explanation and is fairly widely accepted when the track record seems impressive.

4 Bishop Whately’s 1819 *Historic Doubts Relative to Napoleon Buonaparte* was a spoof of excessive skepticism toward the New Testament.

5 Islam tends to portray Ishmael rather than Isaac in this role.
